# Smoking and Mental Health: A Framework for Action in Wales

**DOI:** 10.1192/bjo.2025.10494

**Published:** 2025-06-20

**Authors:** Oliver John, Shane Mills, Adrian Clarke, Alka Ahuja, Dafydd Huw

**Affiliations:** 1 Royal College of Psychiatrists Wales, Cardiff, United Kingdom; 2 NHS Wales, Cardiff, United Kingdom

## Abstract

**Aims:** Smoking contributes to poor mental health and increases inequalities in physical health and premature mortality. Smoking is a leading contributor to the 7–23-year lower life expectancy among people with severe mental illness (SMI) compared with the general population.

In Wales, an estimated 14.1% of people are smokers. Smoking is much more common (33% prevalence) among people with mental health conditions and is even higher among those with SMI (40.5% prevalence).

Welsh Government commissioned NHS Wales’ Joint Commissioning Committee and RCPsych Wales to develop a framework to help reduce smoking rates among people with mental health conditions in Wales.

The framework sought to address three priority areas for action:

Address misperceptions about smoking in mental health settings.

Improve implementation of quitting strategies in mental health settings.

Address the lack of data on smoking and quitting among people with SMI.

**Methods:** In partnership with the Public Mental Health Implementation Centre; a review of data, current interventions, policy and strategy across Wales was undertaken.

This was complemented by consideration of:

Overview of current evidence-based strategies in Wales.

Scaling evidence-based interventions for smokers with mental health conditions and SMI.

Addressing misperceptions among smokers and health professionals.

Upskilling mental health professionals to enable them to motivate and support quit attempts.

**Results:** In addressing the three priority areas for action, the framework focused upon:

Understanding local needs and assets.

Working Together.

Taking action for prevention of smoking, mental health promotion, and reducing inequalities.

Evaluation and measuring outcomes.

Additionally, a deficit in the following areas, led to several recommendations:

Provide training resources for upskilling people working in mental health to support smoking cessation.

Review and augment the implementation strategy for Help Me Quit and the Tobacco Control Delivery Plan to support people with mental health conditions.

Improve accessibility to nicotine replacement therapy (NRT) and other smoking cessation medication. People wanting to quit should have access to more than one quitting aid.

Address data gaps by collecting reporting information on rates of smoking, including among people with mental health conditions.

**Conclusion:** Several next steps are necessary to reduce smoking among people with mental health conditions in Wales:

Develop an implementation strategy for the Tobacco Control Delivery Plan to target people with mental health conditions including SMI.

National campaigns promoting positive mental health should include messages about the mental health harms of smoking.

Major gaps in data on smoking and quitting must be addressed.

